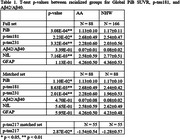# Evaluating impact of racialization on imaging and plasma biomarkers

**DOI:** 10.1002/alz.092968

**Published:** 2025-01-09

**Authors:** Alexandra Gogola, Xuemei Zeng, Anum Saeed, Brian J Lopresti, Beth E. Snitz, Dana Tudorascu, Davneet S Minhas, Milos D Ikonomovic, Julia Kofler, Cristy Matan, Neale S Mason, Tharick A. Pascoal, Howard J Aizenstein, Chester Mathis, William E Klunk, Henrik Zetterberg, Kaj Blennow, Oscar L. Lopez, Steven E. Reis, Victor L Villemagne, Thomas K Karikari, Ann D Cohen

**Affiliations:** ^1^ University of Pittsburgh, Pittsburgh, PA USA; ^2^ Department of Bioengineering, University of Pittsburgh, Pittsburgh, PA USA; ^3^ University of Gothenburg, Mölndal, Gothenburg Sweden; ^4^ University of Gothenburg, Gothenburg Sweden

## Abstract

**Background:**

Imaging and plasma biomarkers are widely used in Alzheimer’s disease (AD) observational studies and clinical trials. Due to the lack of racial or ethnic diversity in earlier studies, a more complete understanding of biomarker differences across racialized groups is needed. Further, results from the few previous studies have disagreed on both the magnitude and direction of AD biomarker differences in racialized groups. We evaluated differences in plasma biomarker and Aβ PET outcomes between participants racialized as either Black or African American (AA) or non‐Hispanic white (NHW).

**Method:**

We evaluated 321 participants enrolled in observational studies at the University of Pittsburgh with PET imaging and plasma biomarker assessments including: Global PiB SUVR, plasma Aβ42/Aβ40, p‐tau181, p‐tau217, p‐tau231, NfL, and GFAP (Simoa HD‐X). Outlier analysis resulted in a total of 88 AA and 166 NHW participants. To further refine the comparison, AA and NHW participants were matched based on age, sex, and ApoE status, resulting in a matched set with 88 AA and 88 NHW participants. A second matched subset was established based on the availability of p‐tau217 measures. Group differences were assessed using Mann‐Whitney U tests.

**Result:**

In both the full and matched sets, Aβ42/Aβ40, NfL, and GFAP showed no differences between the AA and NHW groups (p>0.05). Conversely p‐tau181 and p‐tau231 were higher in AA participants (p<0.05) and global PiB SUVRs and p‐tau217 were higher in NHW participants (p<0.01). (Table 1)

**Conclusion:**

In both the full and matched datasets, the AA and NHW groups showed significant differences in Global PiB SUVR and p‐tau217 outcomes in the opposite direction of p‐tau181 and p‐tau231 outcomes. As such, racialization should be given more consideration in AD clinical research, particularly when biomarker results are used for inclusion or exclusion criteria. Future work will explore the influence of racialization on other imaging and plasma biomarkers and their relationship with cognitive performance.